# The Potential Liver, Brain, and Embryo Toxicity of Titanium Dioxide Nanoparticles on Mice

**DOI:** 10.1186/s11671-017-2242-2

**Published:** 2017-08-02

**Authors:** Xiaochuan Jia, Shuo Wang, Lei Zhou, Li Sun

**Affiliations:** 1 Tianjin University of Science and Technology, Tianjin, 300457 China; 2 Technical Center for Safety of Industrial Products of Tianjin Entry-Exit Inspection and Quarantine Bureau, Tianjin, 300308 China

**Keywords:** Titanium dioxide, Nanoparticle, Liver toxicity, Brain toxicity, Fetus toxicity

## Abstract

Nanoscale titanium dioxide (nano-TiO_2_) has been widely used in industry and medicine. However, the safety of nano-TiO_2_ exposure remains unclear. In this study, we evaluated the liver, brain, and embryo toxicity and the underlying mechanism of nano-TiO_2_ using mice models. The results showed that titanium was distributed to and accumulated in the heart, brain, spleen, lung, and kidney of mice after intraperitoneal (i.p.) nano-TiO_2_ exposure, in a dose-dependent manner. The organ/body weight ratios of the heart, spleen, and kidney were significantly increased, and those of the brain and lung were decreased. High doses of nano-TiO_2_ significantly damaged the functions of liver and kidney and glucose and lipid metabolism, as showed in the blood biochemistry tests. Nano-TiO_2_ caused damages in mitochondria and apoptosis of hepatocytes, generation of reactive oxygen species, and expression disorders of protective genes in the liver of mice. We found ruptured and cracked nerve cells and inflammatory cell infiltration in the brain. We also found that the activities of constitutive nitric oxide synthases (cNOS), inducible NOS (iNOS), and acetylcholinesterase, and the levels of nitrous oxide and glutamic acid were changed in the brain after nano-TiO_2_ exposure. Ex vivo mouse embryo models exhibited developmental and genetic toxicity after high doses of nano-TiO_2_. The size of nano-TiO_2_ particles may affect toxicity, larger particles producing higher toxicity. In summary, nano-TiO_2_ exhibited toxicity in multiple organs in mice after exposure through i.p. injection and gavage. Our study may provide data for the assessment of the risk of nano-TiO_2_ exposure on human health.

## Background

Nanoscale titanium dioxide (nano-TiO_2_) is widely used in the food industry. It has been used for the production of coated candy, preserved fruits, chewing gum, carbonated drinks, powdered drinks (in unsweetened dosage form or concentrated), milk and dairy products, and other food categories [1, 2]. The concentration of nano-TiO_2_ in food reaches as high as 0.5–9 g/kg [1, 3], and many food products that are claimed nano-TiO2-free contain nano-TiO2 [2]. Nano-TiO_2_ has also been widely used in biomedicine, organic pollutant treatment, materials engineering, and cosmetics [4–6]. However, the safety of nano-TiO_2_ exposure remains unclear.

Studies have shown that nano-TiO_2_ can become enriched and toxic in multiple organs after entering the body through several methods, such as administration via the abdominal cavity or inhalation [7, 8]. Nano-TiO_2_ may be toxic to several types of cell, such as human lymphoblastoid cells and hepatoma cells [9, 10]. It can induce an acute stress reaction in glial cells of mouse brains, leading to neuron damage and dysfunction [11]. The survival rate of neuron cell lines exposed to nano-TiO_2_ particles is significantly decreased in a typical time- and dose-dependent manner [12].

Studies have revealed several mechanisms by which these nanoparticles cause toxicity. Nano-TiO_2_ particles may cause genetic toxicity through changing the structure of molecular complex and the permeability of cell membrane [13–15]. Nano-TiO_2_ may produce oxidative stress. During oxidative stress, reactive oxygen species (ROS), such as hydroxyl radicals, are generated and cause DNA oxidation, generating 8-OHG, leading to errors and mutations in DNA replication [16, 17]. Moreover, ROS may induce inflammation and mutual feed-forward interaction between oxidative stress and inflammation, resulting in DNA damage and cell apoptosis [18, 19]. However, the comprehensive systematic data on the toxicity of nano-TiO_2_ remains limited. Our objective was to reveal the effect and the underlying mechanism of nano-TiO_2_ exposure on human health.

In this study, we evaluated the effect and the underlying mechanism of nano-TiO_2_ exposure using mice models. Our findings showed that nano-TiO_2_ may be enriched and cause toxicity in several organs such as the liver, kidney, spleen, heart, lung, and brain through generating an oxidation-reduction imbalance and disorders of gene expression. This may also cause damage to embryonic development. Our study may provide data to assess the potential risk to human health of nano-TiO_2_ exposure.

## Methods

### Chemicals and Reagents

Micro-scale TiO_2_ (micro-TiO_2_) and 5 nm of TiO_2_ in the form of anatase were purchased from Sigma-Aldrich (Shanghai, China), and 10, 60, and 90 nm of TiO_2_ (anatase) were purchased from Run He Ltd. (Shanghai, China). The formaldehyde, nitric acid, hydrogen peroxide, and heparin sodium were reagent grade and were purchased from Sigma-Aldrich (Shanghai, China). Phosphate buffer (PBS), penicillin, and streptomycin were purchased from Gibco (San Diego, USA). Total RNA extraction kits were purchased from Takara (Dalian, China). Reactive oxygen species assay kits were purchased from Jianchen Ltd. (Nanjing, China). Stock TiO_2_ suspension (1%) in Hank’s solution was sterilized at 121 °C for 30 min. The suspension was sonicated and diluted to the desired concentration just before use.

### Animals and Models

For the study of liver and brain toxicity, ICR (imprinting control region) mice (22 ± 3 g, half male and half female) were purchased from the animal center of China Medical University. All experimental procedures involving animals were pre-approved by the Institutional Ethics Committee Tianjin University of Science and Technology and were conducted in accordance with the international guidelines for care and use of laboratory animals. For the study of mouse embryo toxicity, ICR mice (45 females, 20–35 g; 15 males, 35–40 g) were purchased from Beijing Weitong Lihua Ltd. (Beijing, China). All mice were healthy and sexually mature. Five days before treatment, mice were reared in separate cages in a house with good ventilation, a 12-h light/dark cycle, 20 ± 2 °C, 60 ± 10% relative humidity, and ad libitum access to food and water.

Dosing regimen 1 was designed for a general toxicity and brain toxicity test. The mice were randomly divided into six groups and an additional control group, with 10 mice/group. A nanoscale TiO_2_ (nano-TiO_2_) suspension was injected (intraperitoneal (i.p.), 5, 10, 50, 100, 150, and 200 mg/kg) once a day for 14 days. Saline solution was injected into mice of the control group. The mice were observed every day, and no animal died during the study. On the 15th day, blood samples were collected from the orbital sinus. All mice were individually weighed, were anesthetized with 2% Phenobarbital (60 ml/kg, i.p.), and then were sacrificed through cervical dislocation. All tissue samples were collected (brain tissue isolated from cortex and hippocampus) and were stored at −80 °C. Each heart, liver, spleen, lung, and kidney was cut into two portions. One portion was soaked in formalin (10%) solution at 4 °C for pathological examination. The other portion was stored at −20 °C for the determination of the titanium content.

Dosing regimen 2 was designed for the liver toxicity test. Mice were divided into three experimental groups and one control group. Nano-TiO_2_ (5, 10, 50 mg/kg) was administered once per day through gavage for 60 days. The mice in the control group received 0.5% CMC (carboxymethyl cellulose). The mice were observed every day, and no animal died during the study. On the 60th day, the mice were anesthetized with 2% Phenobarbital (60 ml/kg, i.p.), and then were sacrificed through cervical dislocation, the livers were immediately collected and were processed for examination using electron microscopy, determination of ROS and lipid oxidation, and analysis of gene expression.

### Determination of Titanium Content in Target Tissues

A piece of 0.1–0.5-g frozen tissue sample was cut and thawed at room temperature, and then was digested in HNO_3_ (0.5 mL) and H_2_O_2_ (0.5 mL) at 160 °C. After being diluted to 3 mL with 3% nitric acid, the concentration of titanium in the solution was determined using inductively coupled plasma mass spectrometry (ICP-MS). Titanium content in target tissues was then calculated.

### Blood Biochemistry Tests and Calculation of Organ/Body Weight Ratio

The levels of the enzymes in serum samples were analyzed by an automatic biochemistry analyzer (TBA-2000FR, Toshiba, Tokyo, Japan). These enzymes are biomarkers related to the function of the liver and kidney.

The organ/body weight ratio was calculated based on the weights of the organ and the body. The body weights were measured before anesthesia and sacrifice. Organs were weighted after isolation from anesthetized and sacrificed mice.

### Pathological Examination and Transmission Electron Microscopy

Pathological examination of the liver or brain tissues soaked in formalin was performed under a light microscope after hematoxylin staining. For transmission electron microscopy (TEM), the liver tissues were embedded in epoxy resin EPON 812 and were cut into sections as thin as <500 μM after glutaraldehyde and osmic acid fixation. The sections were stained with saturated acetic acid uranium solution (pH 3.5) and lead citrate (pH 12) for 1–2 h. The stained sections were examined using TEM.

### Determination of the Levels of Reactive Oxygen Species, the Activities of Their Metabolic Enzymes, and the Levels of Neurotransmitters

For the liver tissues, the superoxide anion (O_2_^–^) levels were determined using XTT. The activity of catalase (CAT) was determined using OD values at 240 nm, following published procedures [20]. The levels of lipid peroxidation were determined by the content of malondialdehyde (MDA) following published procedures [21].

Brain tissues were homogenized with pre-cooled 1% polyvinylpolypyrrolidone solution (50 mM in pH 7.6 PBS) after isolation. Supernatants were collected after centrifugation at 15,000 rpm for 20 min (Eppendorf 5418, Hamburg, Germany) and were used for subsequent analysis of the activities of superoxide enzyme (SOD), CAT, ascorbate peroxidase (APX), and glutathione peroxidase (GSHPx). SOD activity was determined using NBT (nitro-tetrazolium chloride blue test). Catalase activity was determined using a kit (CAT assay kit, A007-2, Nanjing Jiancheng Bioengineering Institute, Nanjing, China). The activity of APX was measured using a kit (APX assay kit, A123, Nanjing Jiancheng Bioengineering Institute, Nanjing, China). GSHPx activity was determined using a kit (GSHPx assay kit, A005, Nanjing Jiancheng Bioengineering Institute, Nanjing, China). The activities of constitutive nitric oxide synthase (cNOS), inducible nitric oxide synthase (iNOS), and acetylcholinesterase (AChe) were determined using commercial kits (AChe assay kit, A024, Nanjing Jiancheng Bioengineering Institute, Nanjing, China).

The levels of ROS in brain tissues were determined by adding 2′, 7′ dichlorofluorescin diacetate to a final concentration of 10 μM in brain tissue homogenate and by incubating at 37 °C for 30 min; the tissues were then subjected to analysis using flow cytometry.

### Determination of Relative mRNA Levels

Total RNA was extracted from liver tissue samples using a commercial kit (TaKaRa MiniBEST Universal RNA Extraction Kit, 9767, Takara, Dalian, China). Complementary DNA was synthesized using reverse transcription with random primer. The relative mRNA levels of SOD, CAT, GSHPx, MT, HSP70, CYPA, P53, GST, and TF were determined using a real-time quantitative PCR (qPCR) kit (One Step SYBR® PrimeScript™ RT-PCR Kit, PR066A, Takara, Dalian, China). All the primers (Table 1) were synthesized and purchased from Shanghai Sangon Ltd.

**Table 1:** Primers for the gene expression analysis using real-time PCR

Genes	Sequences (5′–3′)	Amplicon (bp)
CAT	Forward: AGCGACCAGATGAAGCAGTG	241
	Reverse: GGGTGACCTCAAAGTATCCAAA	
CYP1A	Forward: CGTCGCAGAGTATCCAGAGG	204
	Reverse: TTAACCGGGTAGCCGTCAAT	
GSHPx	Forward: GGGACTACACCGAGATGAACG	231
	Reverse: TCCGCAGGAAGGTAAAGAGC	
GST	Forward: CCGCTCTTTGGGGCTTTAT	191
	Reverse: GGTTCTGGGACAGCAGGGT	
HSP 70	Forward: CATCGCCAACGACCAGG	162
	Reverse: ACCGCATCGCCGAACTT	
MT	Forward: CTCCTGCACTTGCACCAGC	100
	Reverse: CACATTTGGAGCAGCCCAC	
P53	Forward: GCTGGTTCATCACTCCTCCC	216
	Reverse: GCTTCCCCATTTCACTCTGG	
SOD	Forward: CTGGACAAACCTGAGCCCTAA	242
	Reverse: TCCCCAGCAGCGGAATAA	
TF	Forward: TGTCAGAGCACGAGAATACCAA	224
	Reverse: ATAAAACTCCGCCGCCAC	

### Ex Vivo Embryo Toxicity Test

Embryos of 8.5 embryonic days were isolated from female mice after cervical dislocation and then were cultured in 50.0-mL Hank’s solution containing 3 mL immediately centrifuged serum (ICS) from rats, micro-TiO_2_, or nano-TiO_2_ (0.0, 50.0, 100.0, and 200.0 μg/mL) with 3 embryos in each bottle for 48 h.

To determine the effect of micro-TiO_2_ or nano-TiO_2_ exposure time on embryos, the embryos were cultured in 50.0-mL Hank’s solution containing 3 mL ICS from rats, micro-TiO_2_, or nano-TiO_2_ (200.0 μg/mL) with 3 embryos in each bottle for 16, 26, and 48 h, and then were washed for 48 h with pre-warmed 37 °C Hank’s solution and cultured in 50.0-mL Hank’s solution containing 3 mL ICS from rats.

Embryonic development was evaluated using the Maele-Fabry Van score [22]. The yolk sac diameter, embryonic crown–rump length, head length, and numbers of body sections were examined under a dissecting microscope. The malformation rate of developing embryos was evaluated based on the scores of morphological changes of the forebrain, midbrain, hindbrain, the forelimb bud, the hindlimb bud, the auditory and visual systems, and the heart. More than 10 embryos from 2 ICR mice at embryonic day 10.5 were isolated for control.

### Statistical Analysis

Data was analyzed using SPSS 13 (IBM, Illinois, USA). The difference between the treatment group and the control group was analyzed using a Dunnett’s *t* test. The difference among groups was analyzed using ANOVA. The comparison between two of multiple samples were analyzed using the LSD and SNK tests. Categorical data were analyzed using the chi-square test and rank sum test. If *P* < 0.05, the difference was considered significant.

## Results

### Tissue Distribution of Titanium in Mice After Nanoscale Titanium Dioxide Exposure

**Fig. 1:**
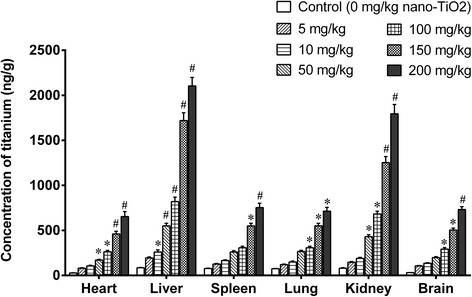
Titanium was accumulated in organs of mice exposed to nano-TiO_2_. Mice were treated with nano-TiO_2_ suspension or saline solution as indicated through intraperitoneal injection once a day for 14 days. * compared to control, *P* < 0.05, # compared to control, *P* < 0.01

We treated mice with nano-TiO_2_ (i.p., 5, 10, 50, 100, 150, and 200 mg/kg) for 14 days and determined titanium contents in the organs of mice. The results revealed that titanium was accumulated in the organs of mice treated with different doses of nano-TiO_2_ (Fig. 1). The magnitude of accumulation was dose-dependent (Fig. 1). The liver was the organ where titanium was enriched most followed by the kidney. The magnitude of accumulation of titanium was approximately the same in the spleen, lung, brain, and heart (Fig. 1). The results suggest that nano-TiO_2_ can be absorbed through GI track and distributed to tissues through the circulatory system and deposited in the organs liver, kidney, spleen, lung, brain, and heart.

### General Toxicity of Nanoscale Titanium Dioxide in Mice

**Fig. 2:**
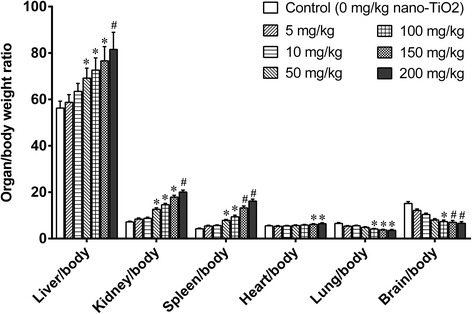
Ratio of organ/body weights in mice exposed to nano-TiO_2_. Mice were treated with nano-TiO_2_ suspension or saline solution as indicated through intraperitoneal injection once a day for 14 days. * compared to control, *P* < 0.05; # compared to control, *P* < 0.01

We treated mice with different doses of nano-TiO_2_ for 14 days and found that there was no difference in body weight gains among groups of mice treated with different doses (data not shown). Low doses of nano-TiO_2_ (5 and 10 mg/kg) did not change the organ/body weight ratio of the liver, kidney, spleen, lung, heart, and brain in mice after i.p. exposure for 14 days (Fig. 2). However, the high doses of nano-TiO_2_ (50, 100, 150, and 200 mg/kg) significantly increased the organ/body weight ratio of the liver, kidney, spleen, and heart and decreased those of lung and brain in mice in a dose-dependent manner (Fig. 2).

**Fig. 3:**
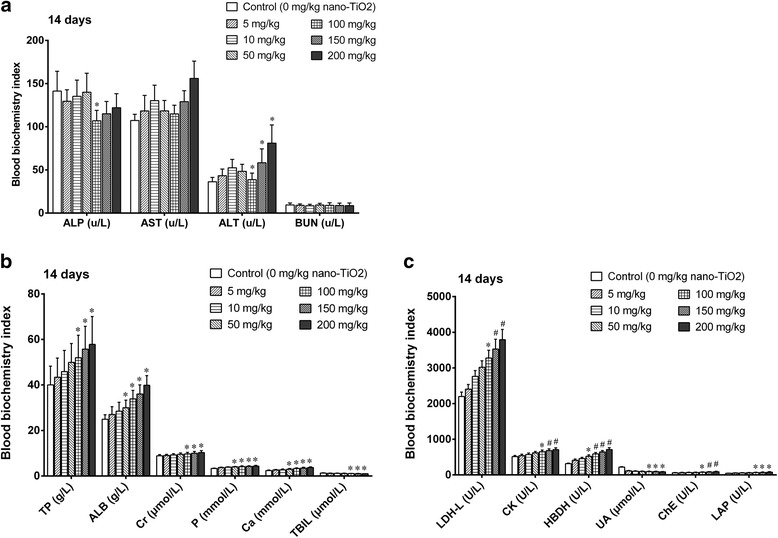
Blood biochemistry index in mice exposed to nano-TiO_2_. Mice were treated with nano-TiO_2_ suspension or saline solution as indicated through intraperitoneal injection once a day for 14 days. * compared to control, *P* < 0.05; # compared to control, *P* < 0.01. **a** Biochemistry index for liver function biomarkers. **b** Biochemistry index for kidney fucntion biomarkers. **c** Biochemistry index for myocardial damage biomarker

Lower doses (5, 10, 50, and 100 mg/kg) of nano-TiO_2_ did not change any blood biochemistry index (Fig. 3). High doses of nano-TiO_2_ (150 to 200 mg/kg) elevated liver function biomarkers alkaline phosphatase (ALP) and alanine aminotransferase (ALT), albumin (ALB), leucine aminopeptidase (LAP), butyrylcholinesterase (PChe), total bilirubin (TBIL), and total protein (TP) levels (Fig. 3). High doses decreased serum uric acid (UA) and blood urea nitrogen (BUN) levels, which are biomarkers for kidney function. They increased serum aspartate aminotransferase (AST), creatine kinase (CK), lactate dehydrogenase (LDH), and alpha hydroxybutyrate dehydrogenase (HBDH) levels, which are indices for myocardial damage (Fig. 3).

These results suggest that high doses of TiO_2_ can cause severe damage to the liver, kidney, heart, and other organs in a dose-dependent manner.

### Liver Toxicity of Nano-TiO_2_ in Mice

**Fig. 4:**
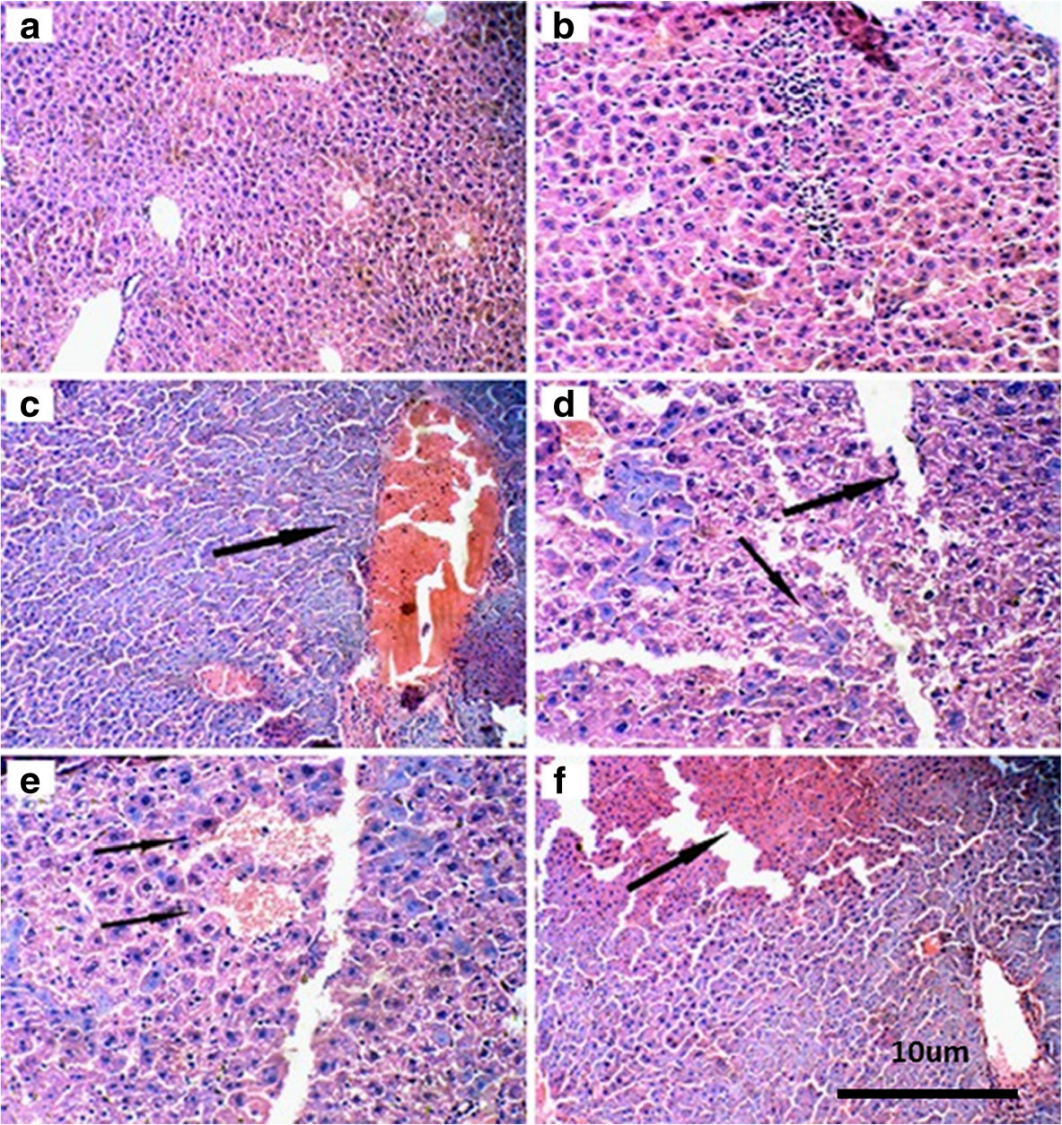
Histology of livers in mice treated with nano-TiO_2_ exposed to nano-TiO_2_. Mice were treated with nano-TiO_2_ suspension or saline solution as indicated through intraperitoneal injection once a day for 14 days. **a** Control. **b** TiO_2_, 5 mg/kg. **c** TiO_2_, 50 mg/kg. **d** TiO_2_, 100 mg/kg. **e** TiO_2_, 150 mg/kg. **f** TiO_2_, 200 mg/kg

We further evaluated the liver toxicity of nano-TiO_2_. Using light microscopy, we found that there was no significant change in the livers of mice exposed to low dose (i.p. for 14 days, 5 mg/kg) nano-TiO_2_ (Fig. 4a, b). We observed marked vascular obstruction and dilation (Fig. 4c, 50 mg/kg), increases in basophils (Fig. 4d, 100 mg/kg), partial ischemia in the liver (Fig. 4e, 150 mg/kg), and obstruction of central veins (Fig. 4f, 200 mg/kg) in mice exposed to nano-TiO_2_ (i.p.).

**Fig. 5:**
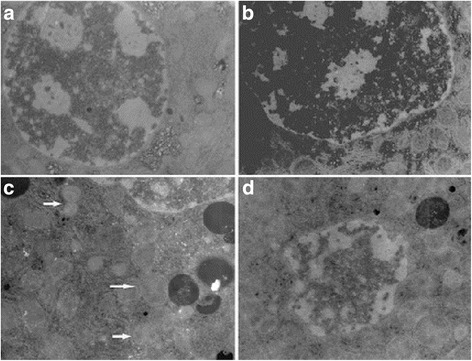
Ultramicroscopic structure of hepatocytes in mice exposed to nano-TiO_2_. Mice were treated with nano-TiO_2_ as indicated through gavage once per day for 60 days. The mice in the control group received 0.5% CMC (carboxymethyl cellulose). **a** Control (×8000). **b** TiO_2_ (5 mg/kg) (×8000). **c** TiO_2_ (10 mg/kg) (×10,000). Arrows indicates mitochondria and vacuoles in mitochondria. **d** TiO_2_ (50 mg/kg) (× 10,000)

However, using TEM, we found a slight swelling of the mitochondria in hepatocytes and presence of condensed chromatin and apoptotic cells in the liver tissues in mice exposed to low dose of nano-TiO_2_ (gavage for 60 days, 5 mg/kg) (Fig. 5a, b). We observed nano-TiO_2_ in the mitochondria of hepatocytes, swelling mitochondria, and vacuoles in mitochondria of the liver cells of mice treated with 10 mg/kg nano-TiO_2_ (gavage for 60 days, Fig. 5c). We further observed nucleolus collapse, scattered chromatin, obvious apoptosis, and/or apoptotic bodies in the liver cells of mice treated with 50 mg/kg nano-TiO_2_ (gavage for 60 days, Fig. 5d). The results indicated that nano-TiO_2_ can lead to pathological damage in liver cells at the subcellular and cellular levels.

**Fig. 6:**
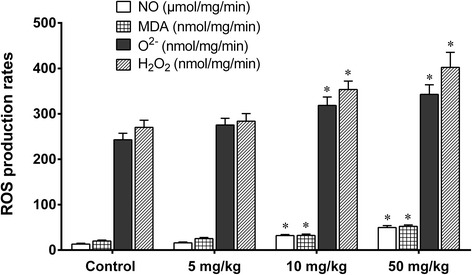
ROS production rates and lipid peroxidation levels in livers of mice exposed to nano-TiO_2_. Mice were treated with nano-TiO_2_ as indicated through gavage once per day for 60 days. The mice in the control group received 0.5% CMC (carboxymethyl cellulose). * compared to control, *P* < 0.05, normalized to total protein

**Fig. 7:**
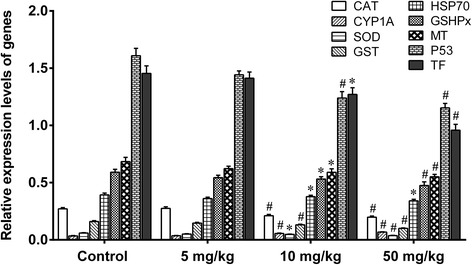
Relative expression levels of genes in livers of mice exposed to nano-TiO_2_. Mice were treated with nano-TiO_2_ as indicated through gavage once per day for 60 days. The mice in the control group received 0.5% CMC (carboxymethyl cellulose). * compared to control, *P* < 0.05; # compared to control, *P* < 0.01, normalized to β-actin

The treatment of mice with 5 mg/kg nano-TiO_2_ for 60 days did not change the levels of ROS such as O^2−^, H_2_O_2_, nitric oxide (NO), and MDA (Fig. 6), or the mRNA levels of SOD, CAT, GSHPx, MT, GST, HSP70, P53, and TF genes in liver tissues (Fig. 7). Treatment of mice with 10 or 50 mg/kg nano-TiO_2_ for 60 days resulted in significant increases in the levels of O^2−^, H_2_O_2_, NO, and MDA (Fig. 6), decreases in the mRNA levels of SOD, CAT, MT, GST, HSP70, P53, TF, and GSHPx genes, and increases in the mRNA levels of CYP1A genes in the livers of mice (Fig. 7). The results showed that high doses of nano-TiO_2_-induced oxidative stress and changes in the expression of protective genes in the livers of exposed mice.

### Brain Toxicity of Nanoscale Titanium Dioxide in Mice

We further evaluated brain toxicity of nano-TiO_2_. We first examined the ratios of brain/body weights in the mice exposed to nano-TiO_2_ (i.p. for 14 days). Low doses (5, 10, 50 mg/kg) did not change the ratios of brain/body weights, and higher doses (100, 150, 200 mg/kg) significantly decreased the ratios of brain/body weights in a dose-dependent manner (Fig. 2). The concentration of Ti in the brain tissues was significantly increased in a dose-dependent manner (Fig. 1).

**Fig. 8:**
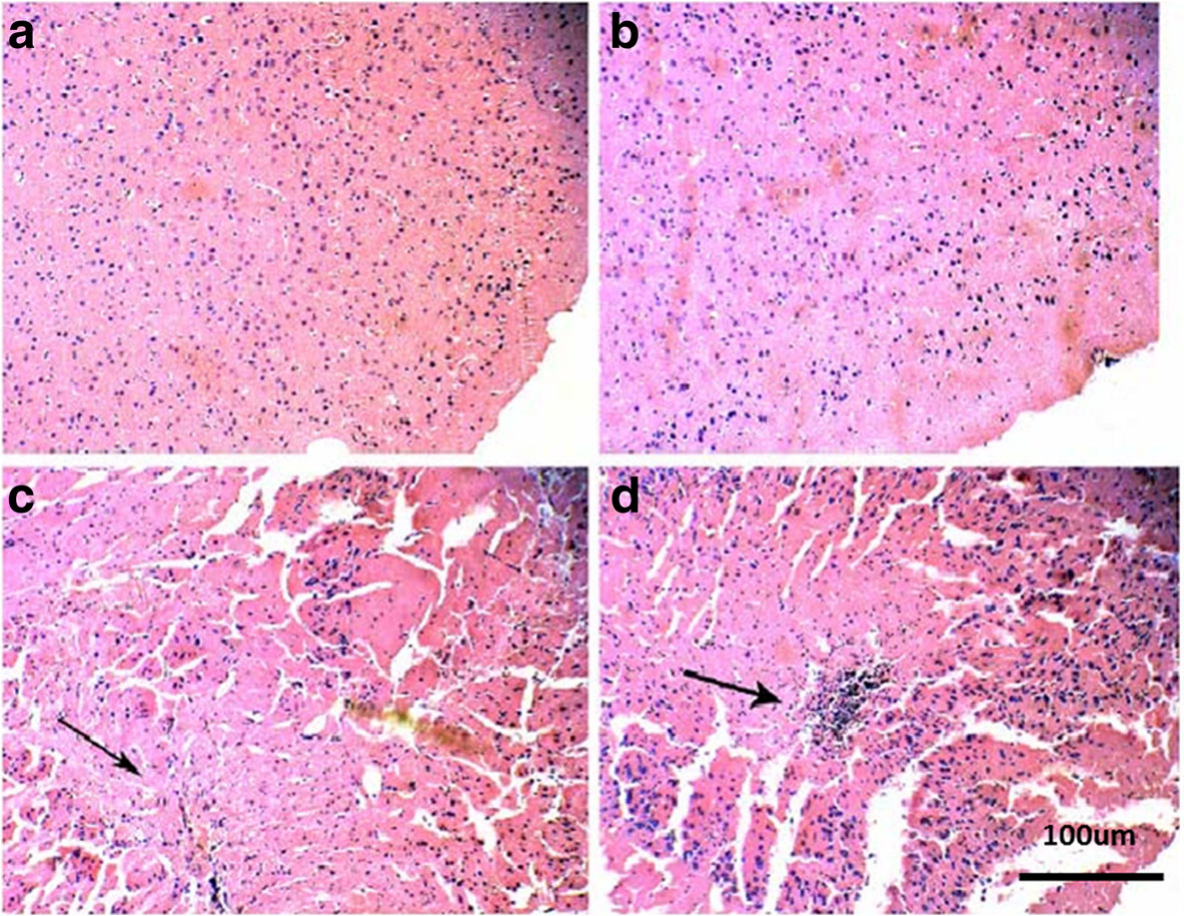
Pathological changes in brain tissue of mice exposed to nano-TiO_2_. Mice were treated with nano-TiO_2_ suspension or saline solution as indicated through intraperitoneal injection once a day for 14 days. **a** Control. **b** 50 mg/kg. **c** 150 mg/kg. **d** 200 mg/kg

We also examined the histological changes in the brain of mice exposed to nano-TiO_2_ (i.p. for 14 days) using hematoxylin staining. We observed that low doses of nano-TiO_2_ (50 mg/kg) did not change the histology of brain tissue in mice after i.p. exposure for 14 days (Fig. 8a, b). Treatment of mice with 100 mg/kg of nano-TiO_2_ resulted in ruptured and cracked nerve cells in the brain tissue (Fig. 8c). Treatment of mice with 150 mg/kg of nano-TiO_2_ resulted in invasion of inflammatory cells in the brain tissue (Fig. 8d). The results showed that high doses of nano-TiO_2_ can cause morphological damage to brain tissue, resulting in an inflammatory reaction.

**Fig. 9:**
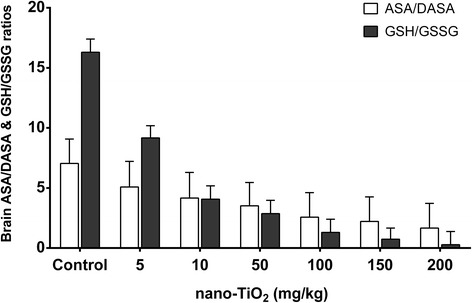
Brain ASA/DASA and GSH/GSSG ratios in the mice exposed to nano-TiO_2_. Mice were treated with nano-TiO_2_ suspension or saline solution as indicated through intraperitoneal injection once a day for 14 days

**Fig. 10:**
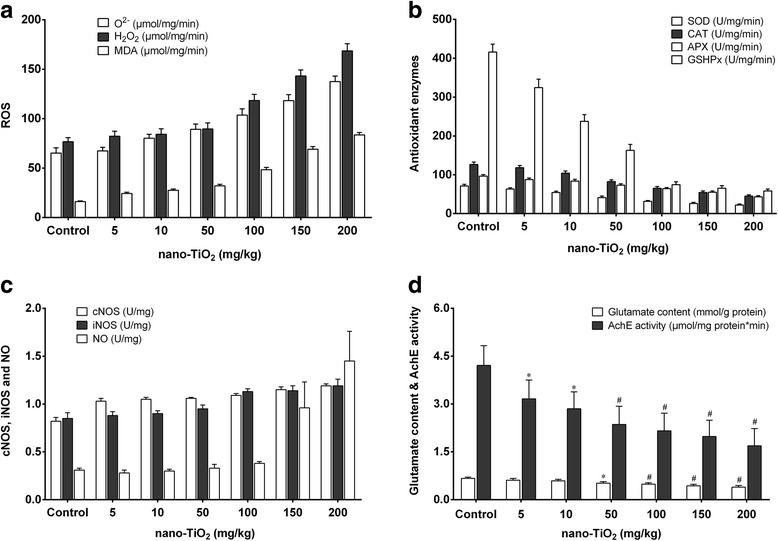
Changes in the ROS, antioxidant enzymes, NO signaling, glutamate, and AchE activities in brains of mice exposed to nano-TiO_2_. Mice were treated with nano-TiO_2_ suspension or saline solution as indicated through intraperitoneal injection once a day for 14 days. *N* = 10, * compared to control, *P* < 0.05; # compared to control, *P* < 0.01. **a** Change of ROS (O2-, H2O2, and MDA) in brains of mice exposed to nano-TiO2. **b** Change of antioxidant enzymes (SOD, CAT, APX, and GSHPx) in brains of mice exposed to nano-TiO2. **c** Change of NO signaling components (cNOS, iNOS, and NO) in brains of mice exposed to nano-TiO2. **d** Change of glutamate content and AchE activity in brains of mice exposed to nano-TiO2

We determined the effects of nano-TiO_2_ on the redox state and signal molecules in brain tissue of mice exposed to nano-TiO_2_ (i.p. for 14 days). We observed that a low dose (5 mg/kg) nano-TiO_2_ did not change O^2−^, H_2_O_2_ and MDA levels, did not change the activities of the antioxidant enzymes APX, CAT, GSHPx, and SOD, or the levels of non-enzymatic antioxidants ASA/DASA and GSH/GSSG. Nor did it change nitric oxide synthase (NOS) activity and NO levels in brain tissues (Figs. 9 and 10). Higher doses of nano-TiO_2_ increased O^2−^, H_2_O_2_, and MDA levels, decreased the activities of the antioxidant enzymes APX, CAT, GSHPx, and SOD, decreased the levels of the non-enzymatic antioxidants ASA/DASA and GSH/GSSG, increased the levels of NO and activities of NOS, and decreased the levels of AchE and blood glucose (GLU) in the brain tissues (Figs. 9 and 10). These results suggest that nano-TiO_2_ may cause damage in the brain in mice after i.p. exposure.

### Toxic Effect of Nano-TiO_2_ on Ex Vivo Mouse Embryos

To evaluate the developmental toxicity of nano-TiO_2_, we first compared the growth and development of in vivo embryos and ex vivo embryos. The results showed that the growth and development of ex vivo embryos was similar to those of in vivo embryos (data not shown). Therefore, we used ex vivo embryos to study the toxic effects of nano-TiO_2_ on embryos.

We investigated the effects of different doses (final concentrations 0.0, 50.0, 100.0, and 200.0 μg/mL) and different exposure times of micro-TiO_2_/nano-TiO_2_ on embryonic growth and development as well as the morphology of tissues and organs through examining the embryonic VXY diameter, crown–rump length, head length, and the number of body sections. The results showed that the micro-TiO_2_ did not change these indicators at any dose (Table 2).

**Table 2:** Effects of TiO_2_ of different sizes at different concentration on the growth and development of mouse in vitro embryos (*N* = 15)

TiO_2_ concentration	VXY diameter	CRL (mm)	Head length (mm)	The number of body sections	Malformation rate (%)	Death rate (%)
micro-TiO_2_					0	0
0.0	4.37 ± 0.08	3.35 ± 0.06	1.45 ± 0.04	30.68 ± 1.65	0	0
50.0	4.36 ± 0.07	3.34 ± 0.13	1.49 ± 0.12	30.46 ± 1.27	20.0*	0
100.0	4.46 ± 0.09	3.39 ± 0.08	1.46 ± 0.11	30.82 ± 1.49	33.3*	0
200.0	4.51 ± 0.11	3.38 ± 0.09	1.46 ± 0.08	30.68 ± 1.29		
5–10 nm						
0.0	4.36 ± 0.07	3.34 ± 0.05	1.46 ± 0.05	30.78 ± 1.65	0	0
50.0	4.37 ± 0.06	3.37 ± 0.8	1.47 ± 0.8	30.63 ± 1.47	0	0
100.0	4.01 ± 0.24*	2.59 ± 0.28*	1.16 ± 0.08#	22.24 ± 3.47*	20.0*	0
200.0	3.21 ± 0.28^#^	2.36 ± 0.49^#^	1.06 ± 0.12^#^	17.68 ± 4.22^#^	33.3*	0
60 nm						
0.0	4.38 ± 0.07	3.35 ± 0.06	1.46 ± 0.06	30.81 ± 1.72	0	0
50.0	4.37 ± 0.06	3.36 ± 0.05	1.47 ± 0.08	30.63 ± 2.14	0	0
100.0	3.41 ± 0.34*	2.51 ± 0.32*	1.22 ± 0.18#	24.17 ± 4.61^#^	6.67	0
200.0	4.32 ± 0.26	2.29 ± 0.51*	0.96 ± 0.21^#^	15.36 ± 4.71^#^	60.0*	0
90 nm						
0.0	4.37 ± 0.07	3.35 ± 0.05	1.46 ± 0.06	30.91 ± 1.67	0	0
50.0	4.38 ± 0.06	3.38 ± 0.11	1.47 ± 0.08	30.13 ± 1.61	0	0
100.0	4.09 ± 0.14*	2.89 ± 0.02*	1.19 ± 0.11^#^	19.17 ± 4.56^#^	52.7^#^	6.67
200.0	3.72 ± 0.21^#^	2.52 ± 0.21^#^	1.08 ± 0.15^#^	14.67 ± 3.57^#^	74.1^#^	26.67*

Compared to control; *P < 0.05, ^#^ *P* < 0.01

For different sizes of nano-TiO_2_, treatment of embryos of 5–10 nm, 60 nm, 90 nm with 50.0 μg/mL TiO_2_ had no effect on embryo VXY diameter, crown–rump length, head length, and the number of body sections (Table 2). Higher doses (100.0 and 200.0 μg/mL) decreased VXY diameter, crown–rump length, head length, and number of body sections, and increased malformation rate (Table 2). For the same dose, there was no obvious difference among groups treated with different sizes of nano-TiO_2_, 50.0, or 100 μg/mL. Treatment of embryos with 200 μg/mL nano-TiO_2_ significantly decreased VXY diameter, crown–rump length, head length, and the number of body sections of mice embryos with the increasing the size of nano-TiO_2_ particles (Table 2).

Treatment of mice embryos with micro-TiO_2_ (200.0 μg/mL) for 16, 24, and 48 h did not change VXY diameter, crown–rump length, head length, and the number of body sections (Table 3). Treatment of mice embryos with nano-TiO_2_ (5–10 nm and 60 nm, 90 nm, 200.0 μg/mL) for 16 h also did not change VXY diameter, crown–rump length, head length, and the number of body sections (Table 3). However, the treatment of mice embryos with nano-TiO_2_ (5–10 nm and 60 nm, 90 nm, 200.0 μg/mL) for 24 and 48 h decreased VXY diameter, crown–rump length, head length, the number of body sections, and increased the malformation rate (Table 3). For the same exposure time, there was no difference in VXY diameter, crown–rump length, head length, the number of body sections, or malformation rate among groups of different sizes of nano-TiO_2_ particles (Table 3).

**Table 3:** Effects of TiO_2_ of different sizes at different exposure time on the growth and development of mouse in vitro embryos

DOE (h)	Embryos (n)	VXY diameter	CRL (mm)	Head length (mm)	The number of body sections	Neubert scores	Malformation rate (%)
Micro-TiO_2_							
0	15	4.38 ± 0.08	3.35 ± 0.09	1.45 ± 0.04	30.76 ± 1.21	54.36 ± 1.32	0
16	12	4.39 ± 0.11	3.35 ± 0.13	1.47 ± 0.12	30.81 ± 1.13	54.14 ± 1.16	0
26	12	4.36 ± 0.15	3.36 ± 0.12	1.49 ± 0.11	30.62 ± 1.26	53.28 ± 1.08	0
48	15	4.41 ± 0.13	3.38 ± 0.11	1.49 ± 0.08	30.42 ± 1.22	53.45 ± 1.22	0
5–10 nm							
0	15	4.37 ± 0.06	3.35 ± 0.06	1.46 ± 0.06	30.86 ± 1.61	54.43 ± 1.38	0
16	12	4.35 ± 0.04	3.03 ± 0.07	1.47 ± 0.12	30.43 ± 1.32	53.12 ± 1.26	13.33
26	12	3.93 ± 0.07*	2.76 ± 0.05*	1.14 ± 0.07*	25.26 ± 1.31*	41.32 ± 1.38*	75.00^#^
48	15	3.01 ± 0.06^#^	2.08 ± 0.08^#^	0.94 ± 0.08^#^	18.34 ± 1.12^#^	26.24 ± 1.43^#^	94.44^#^
60 nm							
0	15	4.39 ± 0.06	3.36 ± 0.07	1.47 ± 0.08	30.90 ± 1.62	54.13 ± 1.31	0
16	12	4.36 ± 0.04	3.13 ± 0.13	1.22 ± 0.17*	30.63 ± 1.45	52.31 ± 1.26	8.33
24	12	4.05 ± 0.09*	2.77 ± 0.08^#^	1.15 ± 0.06*	27.68 ± 1.30*	41.32 ± 1.48*	66.67*
48	15	3.04 ± 0.11^#^	2.13 ± 0.16^#^	0.94 ± 0.78^#^	19.04 ± 1.07^#^	27.24 ± 1.15^#^	100.00^#^
90 nm							
0	15	4.39 ± 0.06	3.36 ± 0.06	1.47 ± 0.08	30.90 ± 1.62	54.13 ± 1.31	0
16	12	4.37 ± 0.04	3.35 ± 0.08	1.22 ± 0.17*	30.63 ± 1.45	52.31 ± 1.26	0
24	12	4.25 ± 0.06	3.08 ± 0.07^#^	1.15 ± 0.06*	27.68 ± 1.30*	41.32 ± 1.48*	56.67*
48	15	3.34 ± 0.09^#^	2.29 ± 0.05^#^	0.94 ± 0.78^#^	19.04 ± 1.07^#^	27.24 ± 1.15^#^	86.67^#^

*DOE* duration of exposure;

Compared to controls, *P < 0.05; ^#^ *P* < 0.01

In summary, these results indicate that nano-TiO_2_ had toxic effects on the growth and development of mouse embryos in dose-dependent and time-dependent manners. The sizes of the nano-TiO_2_ particles may affect toxicity with a trend of increasing toxicity associated with larger nano-TiO_2_ particles.

## Discussion

Nano-TiO_2_ has been widely used in industry and medicine. However, the safety of nano-TiO_2_ exposure remains unclear. In the present study, we investigated the potential toxicity of nano-TiO_2_, using mice models. We find that nano-TiO_2_ accumulates in the heart, liver, kidney, spleen, lung, and brain of mice after exposure (i.p. injection) in a dose-dependent manner. High doses of nano-TiO_2_ significantly increase the organ/body weight ratios of the liver, kidney, spleen, and heart, and decrease those of the lung and brain in a dose-dependent manner. Moreover, high doses of nano-TiO_2_ significantly increase the levels of ALT, ALP, LAP, PChE, TP, ALB, and TBIL, which are indices for liver function. They decrease the levels of UA and BUN, which are renal function indicators. Further, high doses significantly increase the activities of CK, LDH, AST, and HBDH, and significantly increase the levels of GLU, trigylceride, total cholesterol, and high-density lipoprotein. Low doses of nano-TiO_2_ do not change these biochemical parameters. Our data support that nano-TiO_2_ may be toxic and may affect the liver, kidney, heart, GLU, and lipid metabolism at high doses in a dose-dependent manner.

In the present study, we investigated the mechanism of liver toxicity of nano-TiO_2_. We find that high doses of nano-TiO_2_ may cause swelling of hepatocytes with obvious vacuoles in cells, and nuclear condensation in hepatocytes, and apoptosis and necrosis of hepatocytes in liver tissues. This is consistent with previous studies [7, 23, 24]. After the treatment of mice with high doses of nano-TiO_2_, we find that the levels of CAT, GSHPx, and SOD are significantly decreased, and there is nano-TiO_2_ in the mitochondria of hepatocytes, revealed by TEM. This is consistent with previous studies [7, 25–28] suggesting that nano-TiO_2_ generates excess ROS and reduces the antioxidant capacity of the cells through damaging the mitochondria. This is further supported by observation that nano-TiO_2_ can significantly decrease the mRNA levels of SOD, CAT, GSHPx, MT and HSP70, CYP1A1, p53, GST, and TF genes in the mouse liver. SOD, CAT, GSH PX, and MT are involved in liver cell detoxification, CYP1A1 is involved in toxic-substance metabolism and defense against invasion from harmful substances, and HSP70 and p53 are involved in repairing liver cell DNA damage [10, 29–39]. These findings support that the mechanisms for nano-TiO_2_ liver toxicity are damaging mitochondria, generating ROS, and causing expression disorders of protective genes.

In the present study, we investigated the mechanism of brain neurotoxicity of nano-TiO_2_. We find that high doses of nano-TiO_2_ can produce lipid peroxidation and decrease antioxidant capacity, including SOD, CAT, APX, and GSHPx activities, resulting in oxidative stress, which may damage unsaturated fatty acids and brain tissue [24, 26, 37, 40]. We observed rupture and cracking in nerve cells and the infiltration of inflammatory cells in the brain. We further found that the activities of cNOS and iNOS are increased, and NO is excessively released. Glutamic acid levels and AChe activity are decreased in the brain. This is consistent with the effect of Fe_2_O_3_ nanoparticles on olfactory bulb cells [40] and the effect of nano-TiO_2_ on mouse hippocampal neurons [31, 41]. Glutamate is the most abundant amino acid in excitatory neurotransmitters of the nervous system. It is critical for the brain’s development and function [42]. Acetylcholinesterase is a key enzyme for levels of acetylcholine, which is critical for the function of the peripheral and central nervous systems. Nitric oxide regulates many central nervous functions, such as synaptic plasticity, the sleep–wake cycle, and hormone secretion [43]. Therefore, nano-TiO_2_ may cause oxidative stress and may disrupt orders of neurochemical metabolism in brain tissue and therefore have neurotoxicity in the central nervous system.

We find that the micro-TiO_2_ and low doses of nano-TiO_2_ (5–10 nm and 60 nm and 90 nm) do not exhibit toxicity on ex vivo mouse embryos, while high doses of nano-TiO_2_ (100–200.0 μg/ml) exhibit toxicity on ex vivo mouse embryos, as revealed by evaluation of morphology of exposed embryos. Whole embryo culture is a useful tool to assess the developmental toxicity of chemicals [44, 45]. Previous studies show that exposure of 14-day pregnant mice to a single dose of nano-TiO_2_ in the nasal cavity increase the sensitivity of inflammatory response in F1 generation [46, 47]. Nano-TiO_2_ does not affect white pregnant Kunming mice but inhibits growth, increases the rate of stillbirth, and exhibits developmental toxicity [48]. These studies indicate the presence of the developmental and genetic toxicity of nano-TiO_2_. This is further supported by studies that show cleavage and oxidative damage of DNA by nano-TiO_2_, for example, in Zebra fish [16, 49, 50]. Additionally, another shows an increase in the sister chromatid exchange rates in Chinese hamster ovary cells [51]. Nano-TiO_2_ may also prevent chromosome formation during metaphase in the ovary when TiO_2_ concentration is high [51]. These studies consistently show that exposure to high doses of nano-TiO_2_ is linked with developmental and genetic toxicity. Furthermore, our data indicated that the size of nano-TiO_2_ particles may affect its toxicity, with the trend of increasing toxicity being associated with larger nano-TiO_2_ particles (Table 2).

In the current study, we found that titanium accumulates in a dose-dependent manner in the heart, liver, kidney, spleen, lung, and brain of mice after i.p. injection of nano-TiO_2_. This is consistent with published reports that absorption and distribution of nano-TiO_2_ is dependent on blood circulation. Nanoparticles can be absorbed in mesenchymal cells through being ingested by airway epithelial cells; they can then penetrate into the blood or lymph, thus gradually being distributed to the whole body [52, 53]. It is worth noting that nano-TiO_2_ in the abdomen cavity can be absorbed and transported to the brain by the circulatory system, and nano-TiO_2_ can enter directly into the central nervous system without crossing the blood–brain barrier. This is consistent with previous studies [41, 54]. Nanoparticles can also be absorbed by the terminal nerve cell in the respiratory tract and then be transferred to the ganglion through the axon, eventually entering central nervous cells [8, 55]. Nano-TiO_2_ can be absorbed in the nasal cavity through the olfactory epithelium, and then be transported to other parts of the brain, such as the hippocampus, through the olfactory nerve [41, 54]. Therefore, the brain may be directly exposed to nano-TiO_2_. Damage in the brain may be caused directly or indirectly by nano-TiO_2_.

## Conclusions

Ingested nano-TiO_2_ can be distributed to and accumulated in the heart, brain, spleen, lung, and kidney. It exhibits toxicity and causes disorders of the GLU and lipid metabolism. Nano-TiO_2_ causes liver and brain toxicity mainly through increasing oxidative stress, decreasing antioxidant levels, and changing the expression of the protective genes in the liver. In addition, nano-TiO_2_ has adverse effects on the growth and development of mouse embryos and the morphology of the tissues and organs. The size of nano-TiO_2_ particles may affect their toxicity, with a trend of increasing toxicity being associated with larger nano-TiO_2_ particles. These toxic effects are dose-dependent. Our study may provide data for the assessment of the risk of nano-TiO_2_ exposure on human health.

## Abbreviations

ALB: Albumin
ALP: Alkaline phosphatase
ALT: Alanine aminotransferase
AST: Aspartate aminotransferase
BUN: Blood urea nitrogen
CK: Creatine kinase
cNOS: Constitutive nitric oxide synthases
HBDH: Hydroxybutyrate dehydrogenase
ICR: Imprinting control region
iNOS: Inducible NOS
LAP: Qleucine aminopeptidase
LDH: Lactate dehydrogenase
Nano-TiO_2_: Nanoscale titanium dioxide
PChe: Butyrylcholinesterase
ROS: Reactive oxygen species
TBIL: Total bilirubin
TP: Total protein
UA: Uric acid
